# Early experience with next-generation wireless technology for detecting cochlear implant electrode tip fold-over and placement without X-ray: nucleus SmartNav

**DOI:** 10.1007/s00405-025-09667-4

**Published:** 2025-09-18

**Authors:** Chang-Hee Kim, Bong Jik Kim, Jung Kyu Lee, Byung Yoon Choi

**Affiliations:** 1 Department of Otorhinolaryngology-Head and Neck Surgery, Konkuk University Medical Center, Research Institute of Medical Science, Konkuk University School of Medicine, Seoul, Republic of Korea; 2 Department of Otorhinolaryngology-Head and Neck Surgery, Chungnam National University College of Medicine, Chungnam National University Sejong Hospital, Daejeon, Korea; 3 Department of Otorhinolaryngology-Head and Neck Surgery, Seoul National University Bundang Hospital, Seoul National University College of Medicine, 300 Gumi-Dong, Bundang-Gu, Seongnam, 13620 Republic of Korea; 4 Sensory Organ Research Institute, Seoul National University Medical Research Center, Seoul, Republic of Korea

**Keywords:** Cochlear implant, SmartNav, Tip fold-over, Electrode placement, Slim modiolar electrode

## Abstract

**Purpose:**

To evaluate the effectiveness of the SmartNav technology in detection of the well-known phenomenon of tip fold-over (TFO) during cochlear implantation (CI) using slim modiolar electrode (SME), comparing it to intraoperative X-ray imaging.

**Method:**

In 134 ears with normal anatomy that underwent CI using SME at a tertiary center, SmartNav results were compared to intraoperative X-ray findings, with X-ray used as the gold standard for verifying TFO.

**Results:**

SmartNav identified all 8 TFO cases (100% sensitivity, 99.21% specificity) confirmed by X-ray, including one with subtle distal kinking that X-ray could have missed. Additionally, SmartNav technology was effective in cases with cochlear nerve deficiency and performed reliably under local anesthesia and in revision surgeries.

**Conclusion:**

SmartNav offers a highly sensitive and reliable method for detecting TFO during CI at least in normal cochlear anatomy, dramatically reducing the need for intraoperative X-ray, thereby decreasing anesthesia time and eliminating radiation exposure.

## Introduction

The cochlear implant (CI) is surgically implanted electrically stimulating neural prosthetic device, and have been proven to be an effective rehabilitation option for severe-to-profound hearing loss. The growing use of CIs for hearing rehabilitation has been driven by technical advancement including electrode technology, and correct placement of electrode array within the scala tympani is crucial for better postoperative outcome. The gap between electrode array and auditory nerve fibers may cause shunting of the current, reducing the selectivity of electrical stimulation. In an effort to improve the electrode-nerve interface, the modiolar hugging electrodes have been introduced [1–4]. These electrodes encircle the modiolar wall during insertion, positioning them in the closest vicinity of the spiral ganglion neurons. Although these perimodiolar electrodes ensure more focused excitation, decreased stimulation thresholds and energy consumption, they may cause insertion trauma due to their large size, leading to translocation into the scala vestibuli and electrode kinking or folding [3, 5].

To overcome these drawbacks, thinner, more flexible, and atraumatic slim modiolar electrodes (SMEs) were designed, aiming to minimize cochlear damage while maintaining modiolar proximity. SMEs have been reported to demonstrate advantages in hearing preservation and speech perception [5–7]. However, SMEs are more susceptible to tip fold-over (TFO) because the thinner and more flexible the perimodiolar electrodes, the higher the likelihood of premature unloading before reaching the cochlear curvature [1, 4]. It was reported that the incidence of TFOs was 1.4–6.5% in SMEs compared to 0.005% in lateral wall electrode arrays [8]. TFOs may elicit significant disadvantages, preventing optimal outcomes. The effective insertion length of the electrode is reduced in TFOs, resulting in diminished stimulation of the mid- and low-frequency regions within the cochlea [9, 10], and stimulation of the folded tip leads to a mismatch of tonotopic information and channel interaction [11]. Furthermore, TFOs may cause intrinsic damage to the intracochlear structures and an inflammatory response in the cochlear duct, resulting in intracochlear ossification or a reduced number of spiral ganglion neurons [12, 13]. Inadequate intracochlear excitation in TFOs may potentially causes unexpected issues such as audiological discomfort, vertigo, tinnitus, and co-stimulation of the facial nerve [14, 15].

Thus, intraoperative detection of TFO is crucial for the best postoperative outcome. Among various radiological options including X-ray, cone-beam temporal bone computed tomography (TBCT), and fluoroscopy, X-ray is the most commonly used intraoperative diagnostic tool for verifying electrode placement due to its wide availability, low cost, relatively quick procedure, and low radiation exposure. However, in spite of these advantages over other imaging modalities, the issues regarding the duration of general anesthesia and surgery-related infection may still be a weakness of intraoperative X-ray. In addition to X-ray, electrophysiological measures can also be employed during surgery to offer real-time feedback on electrode arrays. CI manufacturers provided protocols for measuring electrically stimulated action potentials to determine the functionality of implanted device and auditory nerve responses to the stimulus. Although these measures can objectively evaluate individual electrode responses during surgery and confirm the functionality of the array, they cannot solely ensure the array's correct placement.

The Cochlear™ Nucleus® SmartNav System, a wireless intraoperative measurement tool using electrode voltage telemetry (EVT) was introduced in 2022. This system employs a sterile-dressed processor connected via Bluetooth to an Apple iPad, and conducts a placement check of electrode arrays using a proprietary trans-impedance matrix (TIM) algorithm. To investigate the proper electrode positioning, SmartNav System analyzes electrical currents between intracochlear and extracochlear electrodes. The TIM is produced by repeated measurements at each electrode contact, and the TIM displays a decrease in intracochlear potential as the distance from the stimulating electrode increases in case of a correctly positioned electrode array. When TFO occurs, the TIM reveals a secondary peak in intracochlear potential [16].

This study investigated the usefulness of SmartNav in detecting the presence of TFO intraoperatively compared to conventional X-ray measurement and also evaluated whether the use of Nucleus SmartNav would also be applicable in cases such as revision surgeries, local surgeries or cochlear nerve deficiency.

## Patients and methods

### Subjects

Among all 145 consecutive ears where CI was performed using the CI632 electrode at a single tertiary referral center between July 2024 and January 2025, cases with cochlear anomalies (*n* = 11) were excluded. Resultantly, a total of 134 consecutive ears from 98 patients (38 men and 60 women, 5 months–76 years old, with a mean age of 29.3 years), were included in this study. There were 36 bilateral implants and 62 unilateral implants. There were 65 cases of CI performed on the right side and 69 cases on the left side, respectively. The procedures were performed by a single surgeon, and Cochlear Nucleus CI632 (Cochlear Limited Cochlear Headquarters, Australia) were used in all cases. Only patients with normal appearance of cochlea on preoperative imaging studies were included in the present study.

### Intraoperative methods and data analysis

The CP1150S Surgical Processor was wrapped in a sterile vinyl bag, and positioned on the patient’s skin directly over the receiver-stimulator. Then, the CP1150S Surgical Processor was paired to Nucleus SmartNav System via Bluetooth, and SmartNav Live Diagnostics were activated. The electrode arrays were inserted via a conventional round window or extended round window approach, and SmartNav System recorded electrode insertion speed.

After the completion of the electrode insertion, all patients underwent intraoperative x-ray scans in the operating room to verify the electrode tip placement, as recommended [17, 18]. The presence of TFO was determined based on the initial X-ray images. In cases with TFO at the initial X-ray, the electrode was retracted, reloaded, and reinserted until confirmation of correct electrode placement in the final X-ray scans. The impedance, electrode placement and electrically evoked compound action potential (ECAP) were checked using SmartNav system. When measuring ECAP responses, referred to as ‘neural response telemetry (NRT)’ using SmartNav technology, if responses could not be detected on more than two channels, the conventional method was also employed to measure ECAP responses to validate the efficacy of ECAP measurement of SmartNav technology. Statistical analysis was performed using IBM SPSS Statistics Program (Ver. 29.0, IBM). This study was approved by the Institutional Review Board of Seoul National University Bundang Hospital (No. B-2503–958-103).

## Results

It was possible to verify ECAP responses and to check electrode placement using SmartNav technology for all 134 ears (98 patients) with normal cochlear anatomy where CI632 was inserted. Four cases which underwent CI even under local anesthesia tolerated SmartNav measurement. During impedance checks using SmartNav, open circuits were detected in four ears. In these cases, the conventional impedance check method was used to reverify the presence of open circuits, and the same results were confirmed. Open circuit was resolved by reloading followed by reinsertion in 2 ears and replacing them with backup devices in 2 ears.

**Fig. 1:**
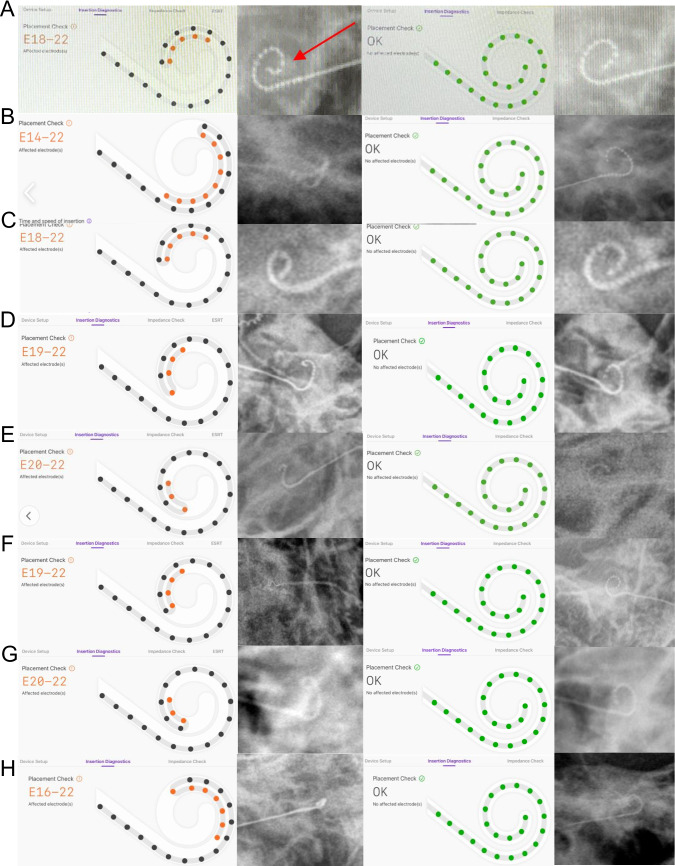
A tip fold-over (TFO) detected by SmartNav system. Eight cases (**A** to **H**) are demonstrated. A TFO was detected in the initial SmartNav system (first panels, **A** to **H**), which is confirmed by X-ray images (second panels, **A** to **H**). After reloading and reinsertion, correct placement of electrodes within the cochlea is verified by both SmartNav (third panels, **A** to **H**) and X-ray images (fourth panels, **A** to **H**). Note that a tiny kinking of the distal electrode (red arrow, second panel of **A**), which might have been overlooked when relying solely on X-ray for detection, is detected by SmartNav system (first panel of **A**). For the second panel of Fig. 1F, we acknowledge that the resolution is suboptimal. To enhance visual clarity, particularly in the electrode channel regions, we have added white dots to mark the positions of individual channels

**Fig. 2:**
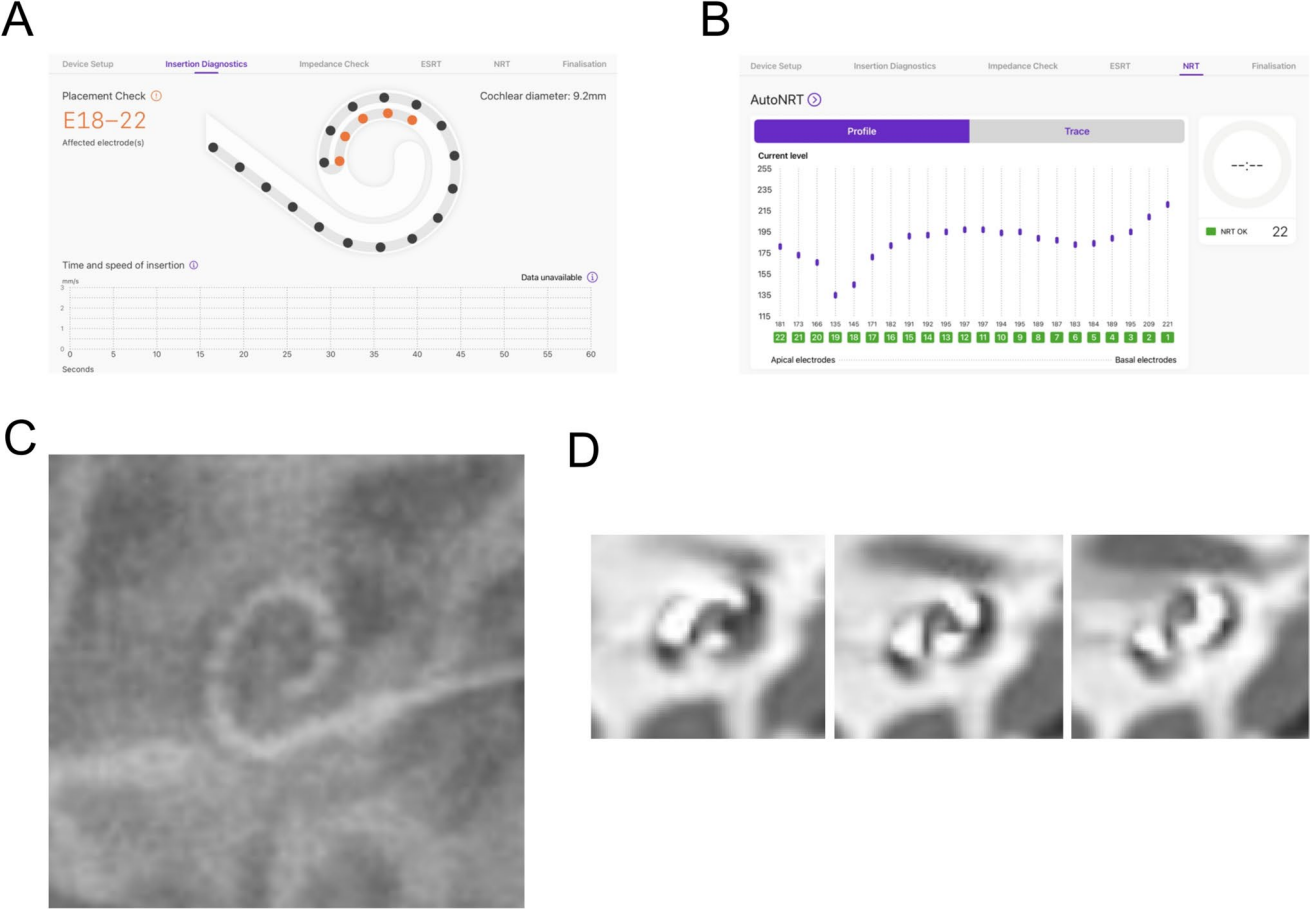
A case of false positive tip fold-over (TFO) in the SmartNav system. (**A**) The electrode placement check by SmartNav demonstrates TFO. (**B**) The NRT measurement shows normal ECAP in all 22 electrode channels. (**C**) The X-ray image reveals correct spiral curve of electrode array within the cochlea. (**D**) Coronal views of postoperative TBCT demonstrate correct positioning of electrode array

Regarding the detection of TFOs, the results of SmartNav were compared to X-ray images for each case with X-ray consistently used as the gold standard for determining TFO. In SmartNav, a TFO was defined as an abnormal finding in the placement check results, specifically when channel 22 was not positioned distal to channel 21 or channel 20 along the trajectory. Then, as previously described [17], we performed intraoperative X-ray scans on all patients in the operating room to confirm the position of the electrode tip. The detailed procedure is as follows: After electrode insertion, a placement check is first performed using SmartNav. Regardless of the SmartNav results, an intraoperative X-ray is subsequently taken. ECAP measurements are conducted only if both the SmartNav assessment and intraoperative X-ray yield normal findings. If a TFO is identified during the SmartNav placement check and this finding is confirmed on the intraoperative X-ray, the electrode is withdrawn, reloaded, and reinserted. A repeat placement check is then performed, and if the findings are normal, a follow-up intraoperative X-ray is used to confirm that the TFO has been corrected. Only then is ECAP measurement performed. Conversely, if the intraoperative X-ray shows no evidence of TFO and appears normal, ECAP measurements proceed without reinsertion. Among 134 ears, X-ray images verified electrode TFO in 8 cases (6.0%; Table 1). Of 8 cases with TFO, 4 were right-sided and 4 were left-sided implants. The TFO was also identified by SmartNav in all 8 ears. In one case, SmartNav successfully detected even the mildest form of TFO, tiny kinking of the distal electrode (Fig. 1A), which might have been easily overlooked without its assistance, even with a very meticulous review of the X-ray alone. The other 7 cases showed a classic TFO (Fig. 1B to H). In all of these 8 cases with TFO, reloading and reinsertion of the electrodes was performed in a way previously suggested to correct TFO [17]. Correction of TFO of electrodes was also verified by both SmartNav and X-ray image after reinsertion (Figs. 1A to H, third and fourth panels). In one patient, while the SmartNav showed TFO (Fig. 2A), X-ray image revealed normal configuration of the electrode (Fig. 2C), which was confirmed by postoperative coronal view of TBCT (Fig. 2D) (Table 1). Resultantly, the sensitivity and specificity of SmartNav for detection of TFO in the cochlea with normal anatomy was 100% (95% CI: 67.56% - 100%, power: 38.9%) and 99.21% (95% CI: 95.64% - 99.86%, power: 66.1%), respectively. The positive predictive value (PPV) of the SmartNav was 88.9% (power: 26.5%) and the false negative rate (FNR) was 0%.

**Table 1:** Comparison of SmartNav to X-ray for detection of electrode tip fold-over

		SmartNav (*n* = 134)	
		Normal	Tip fold-over
X-ray (*n* = 134)	Normal	125	1
	Tip fold-over	0	8

**Fig. 3:**
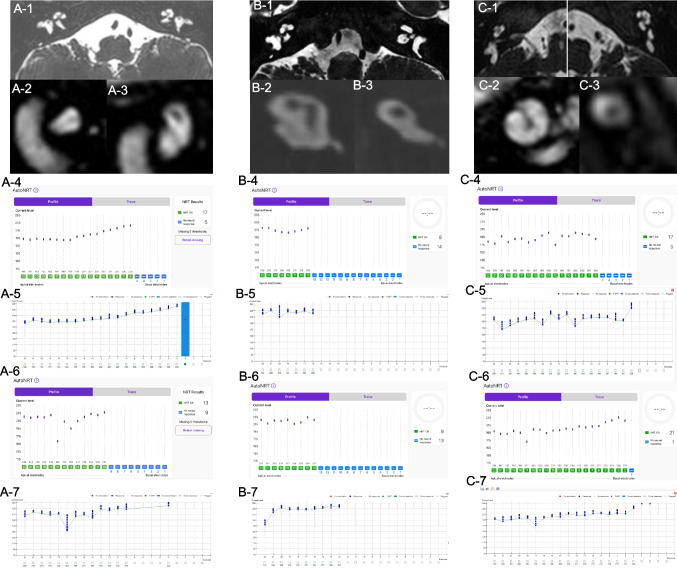
The AutoNRT by SmartNav system in 3 patients (**A**, **B** and **C**) with bilateral cochlear nerve deficiency. Axial views of proton density internal auditory canal MRI reveal bilateral cochlear nerve deficiency, demonstrating the space normally occupied by the nerve is replaced with cerebrospinal fluid (A-1, B-1 and C-1). Sagittal views of T2-weighted internal auditory canal MRI demonstrate cochlear nerve deficiency in the right sides (A-2, B-2 and C-2) and the left sides (A-3, B-3 and C-3). The AutoNRT measurements by SmartNav system and conventional NRT measurements in the right ear reveal normal ECAP responses in 17 (A-4) and 19 (A-5) out of 22 channels, 8 (B-4) and 7 (B-5) out of 22 channels, and 17 (C-4) and 18 (C-5) out of 22 channels, respectively. The AutoNRT measurements by SmartNav system and conventional NRT measurements in the left ear reveal normal ECAP responses in 13 (A-6) and 14 (A-7) out of 22 channels, 9 (B-6) and 10 (B-7) out of 22 channels, and 21 (C-6) and 18 (C-7) out of 22 channels, respectively

There was no significant difference in impedance between cases with and without TFO; therefore, prior to identifying TFO through the placement check, no abnormality could be detected based on impedance alone. When a TFO was identified during the SmartNav system’s placement check, we performed X-ray confirmation and subsequent reinsertion and correct it. Only after confirming successful correction of a TFO, NRT measurements using SmartNav were conducted. Intraoperative NRT measurement was precisely conducted using the SmartNav system in all 134 ears including six ears with cochlear nerve deficiency. The results of ECAP measurements from six ears with cochlear nerve deficiency indicate that there is no significant difference in overall trends between the SmartNav method and the conventional method (Fig. 3). The difference in time required for NRT measurements between the conventional method and SmartNav was notably dramatic in cases of cochlear nerve deficiency cases. While the conventional method required over 10 min on average for these patients (Mean: 11 min 18 s), SmartNav completed the measurements in just over 3 min (Mean 3 min), demonstrating that the latter is significantly more efficient for NRT assessment (*p*-value: 1.71 × 10^−5^, Welch's t-test).

The insertion speed of CI632 electrode was available for all of 7 cases with TFO and 113 cases without TFO. The average insertion speed was 1.38 ± 1.10 mm/s (range, 0.12 mm/s - 3.43 mm/s) in 7 cases with TFO and 0.97 ± 1.10 mm/s (range, 0.01 mm/s – 4.74 mm/s) in 113 cases without TFO, which was not significantly different (*p* = 0.356, Mann–Whitney U test). We identified 8 outlier cases in our patient cohort that exhibited an exceptionally slow insertion speed of 0.01–0.12 mm/s.

## Discussion

The present study, to the best of our knowledge, is the study on the effectiveness of SmartNav technology that includes the largest number of cases. Another advantage of this study is its homogeneity, as it exclusively used the slim modiolar CI632 electrode. This study found that SmartNav demonstrated exceptional effectiveness in detecting TFO through placement checks intraoperatively in cases with normal anatomy cochlea. In detecting TFO, the sensitivity, specificity, PPV, and FNR of SmartNav was 100% (95% CI: 67.56% - 100%), 99.21% (95% CI: 95.64% - 99.86%), 88.9%, and 0%, respectively. The excellent performance of SmartNav in the present study was consistent with the results of previous study [19]. Considering that one case with a very tiny distal kinking was detected by SmartNav in this study, it seems that SmartNav sensitively detects TFO by identifying any situation where electrode 21 or 22 is not perceived to be positioned ahead of electrode 20 along the pathway, no matter how slight, as an indicator of TFO to enhance its detection sensitivity. This strategy enabled the systematic screening of subtle TFOs (which might otherwise have been overlooked or undetected on X-ray), regardless of their functional outcomes. The SmartNav system appears to have been configured with a focus on sensitivity over specificity, allowing for highly sensitive screening for TFO detection. Consequently, there was one instance in this study where a TFO was falsely identified, despite its absence, resulting in a positive predictive value (PPV) of 88.9%. This suggests that when a TFO is detected using SmartNav, it is essential to confirm its presence through X-ray imaging before proceeding with reloading and reinsertion processes.

Cooper et al. suggested that the potential benefits of SmartNav over X-ray may be decrease in anesthesia time, lack of radiation exposure specially in simultaneous bilateral CIs in infants for both patients and medical providers, and enhanced reliability [19]. In that study, SmartNav proved to be more straightforward to interpret compared to traditional X-ray, while maintaining similar reliability. Because the quality of intraoperative imaging used to evaluate electrode placement can differ depending on the technician's expertise and the patient's anatomical features especially for patients receiving bilateral CI, the identification of TOF using SmartNav may be more rapid and unambiguous. It was reported that SmartNav placement check required a significantly less time (2.12 min) compared to X-ray imaging (14.23 min), reducing total anesthesia time by using SmartNav [19]. Moreover, as demonstrated in our study, the findings that reliable SmartNav results were obtained even in cases performed under local anesthesia and that SmartNav could be used without any issues in revision cases, can be considered additional benefits of SmartNav. In addition, SmartNav measurements were successfully obtained even under challenging conditions, such as cochlear nerve deficiency, with results that were comparable to conventional ECAP measurements. In particular, for infants under one year of age with cochlear nerve deficiency, where prolonged anesthesia time may pose a burden on the developing brain [20], the use of SmartNav in bilateral simultaneous surgeries offers significant advantages. Beyond reducing the time required for X-ray imaging, it also substantially decreases the time needed for ECAP response measurements. These benefits highlight the enhanced utility of SmartNav in such cases.

Our results suggest that by using SmartNav, surgeons may efficiently detect, correct, and reassess positioning issues, particularly TFO, with minimal time investment and without subjecting the surgical staff to additional radiation exposure. However, the SmartNav system currently does not provide a platform for calculation of the angular insertion depth (AID) as for the CI632 electrode, and it is believed that providing this measurement would be beneficial since AID could affect modiolar proximity even under CI632 use especially in case of short CDL [21]. Moreover, although the SmartNav system demonstrated high diagnostic performance in detection of TFOs, the statistical power was limited for sensitivity (38.9%) and PPV (26.5%) due to the small number of TFO-positive cases. In contrast, specificity showed a moderate power of 66.1%, given the larger sample of TFO-negative cases. These findings suggest that while initial results are promising, further validation with larger cohorts is warranted. Another potential technical limitation is that the SmartNav system's reliance on an internet connection may pose challenges in operating rooms where connectivity is unreliable, such as those in hospital basements. This could significantly impact its implementation in certain medical centers. Nevertheless, technically speaking, the functionalities of SmartNav—including impedance measurement, placement check, ECAP measurement, and insertion speed monitoring—do not require an internet connection. Internet access is only needed for logging in and subsequently retrieving and uploading data to the cloud. However, even these steps can be bypassed by utilizing the'Limited Functionality Mode,'which allows full use of SmartNav features without the need for internet access or login. In some cases, we observed an exceptionally slow speed of insertion (SOI), which is likely due to inaccurate measurement rather than actual slow insertion. According to the manufacturer's technical perspective, their SmartNav support team reviewed the raw data and provided the following explanation: The SOI algorithm was originally developed and validated under the assumption that a ‘dry’ electrode is inserted into a fluid-filled cochlea. The algorithm relies on detecting the ‘wetting’ of the electrode contacts upon entering the perilymph. If the electrode is pre-wetted—either through deliberate hydration, residual blood, or excess fluid in the surgical field—the expected wetting signal may be altered, potentially compromising the algorithm’s performance. Based on this understanding, the unexpectedly low insertion speeds observed in the reviewed cases may be attributed to: (1) a pause in insertion prior to the basal electrode (E1) becoming adequately wet, which could have led to a prolonged insertion time being recorded, and (2) the routine surgical practice of applying steroids to the electrode and round window niche, which may result in premature wetting. These factors, while clinically motivated to preserve residual hearing, may fall outside the operating parameters assumed by the current algorithm. The manufacturer acknowledges that such discrepancies are likely due to surgical conditions not currently accounted for by the algorithm and suggests that future iterations may need to incorporate greater flexibility to accommodate diverse intraoperative scenarios.

## Conclusion

This study represents the largest case series to date evaluating the effectiveness of SmartNav technology, demonstrating its high sensitivity and reliability in detecting TFO during CI surgeries with the slim modiolar CI632 electrode. SmartNav achieved exceptional diagnostic performance, with sensitivity, specificity, and FNR values of 100%, 99.21%, and 0%, respectively, highlighting its ability to systematically screen for subtle TFOs, even those undetectable on X-ray. The system's high sensitivity, coupled with its time-saving benefits, reduced anesthesia duration, and elimination of radiation exposure, further underscores its clinical utility, particularly in complex scenarios such as bilateral surgeries or cases involving cochlear nerve deficiency. Overall, SmartNav potentially offers a valuable and efficient alternative to traditional X-ray imaging, providing surgeons with a precise and streamlined tool for intraoperative electrode placement assessments, at least in normal anatomy cochlea. Since false positives—instances where SmartNav detects TFO without actual translocation—may occur in the detection of TFOs, intraoperative X-ray imaging is likely to be used more selectively in the future, especially when SmartNav indicates a TFO in cochleae with normal anatomy.
